# Supplementary material for: Unusual flexibility of transparent poly(methylsilsesquioxane) aerogels by surfactant-induced mesoscopic fiber-like assembly
Source: Nat Commun. 2024 Jan 11;15:461. doi: 10.1038/s41467-024-44713-5 (PMC10784555; doi:10.1038/s41467-024-44713-5)
Supplement: Supplementary file 1 — Supplemantary Information [file 41467_2024_44713_MOESM1_ESM.pdf]

Supplementary Information

Unusual Flexibility of Transparent Poly(methylsilsesquioxane) Aerogels by Surfactant-Induced Mesoscopic Fiber-like Assembly

Ryota Ueoka,[a,‡] Yosuke Hara,[a,‡] Ayaka Maeno,[b] Hironori Kaji,[b] Kazuki Nakanishi,[c,d] and Kazuyoshi Kanamori[a]*

---

a. Department of Chemistry, Graduate School of Science Kyoto University Kitashirakawa, Sakyo-ku, Kyoto 606-8502 (Japan)

b. Institute for Chemical Research, Kyoto University, Gokasho, Uji-city, Kyoto 611-0011 (Japan)

c. Institute of Materials and Systems for Sustainability Nagoya University Furo-cho, Chikusa-ku, Nagoya, Aichi 464-8601 (Japan)

d. Institute for Integrated Cell-Material Sciences, Kyoto University Yoshida, Sakyo-ku, Kyoto 606-8501 (Japan)

---

‡ These authors contributed equally to this work.

* Corresponding author, E-mail: kanamori@kuchem.kyoto-u.ac.jp

Supplementary Figures, Tables and Movies

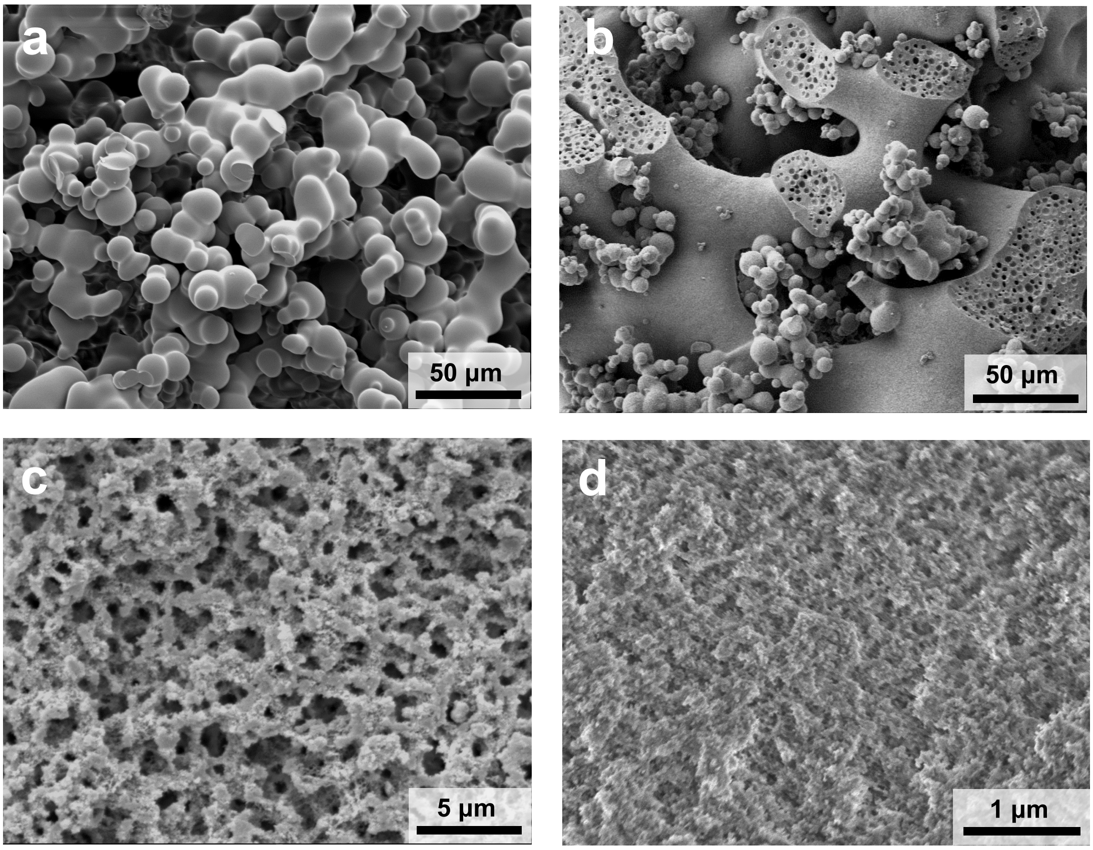

Supplementary Figure 1. FE-SEM images of the PMSQ aerogels prepared with varied amount of F127; (a) 0 g, (b) 0.32 g, (c) 0.36 g and (d) 0.40 g.

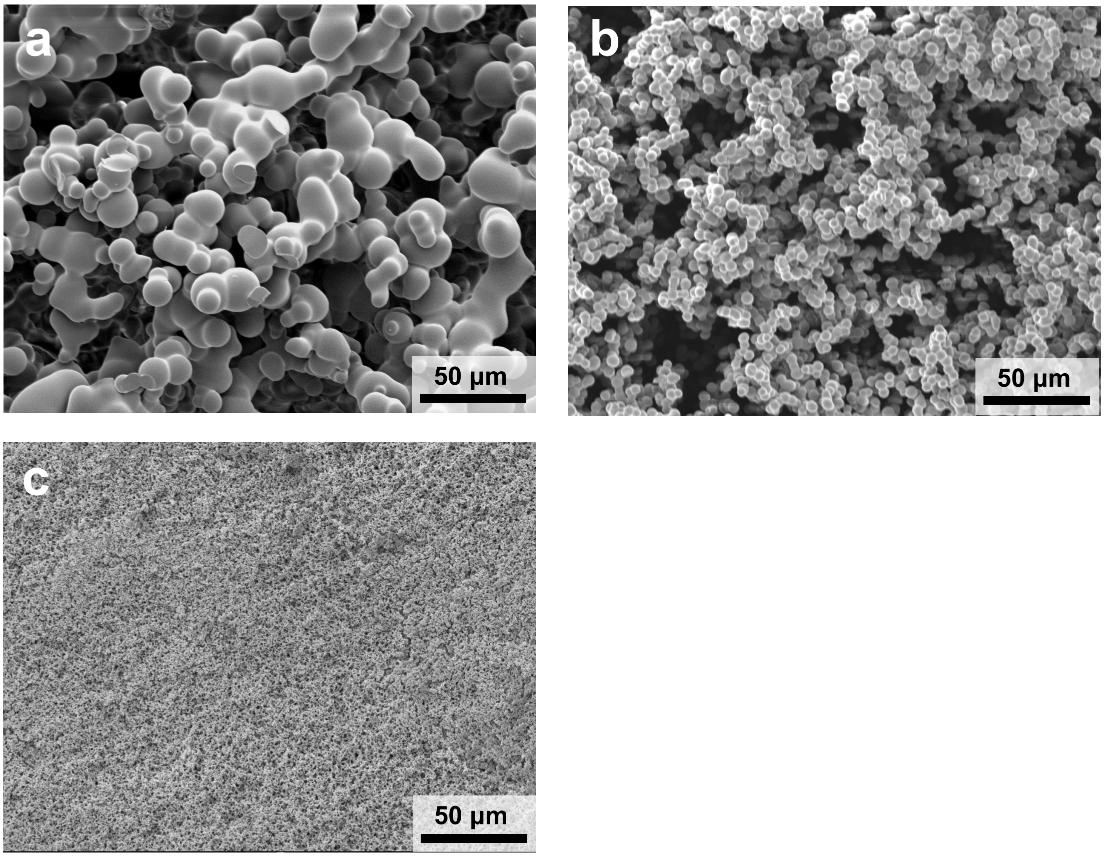

Supplementary Figure 2. FE-SEM images of the PMSQ aerogels prepared with varied amount of CTAC; (a) 0 g, (b) 0.01 g and (c) 0.03 g.

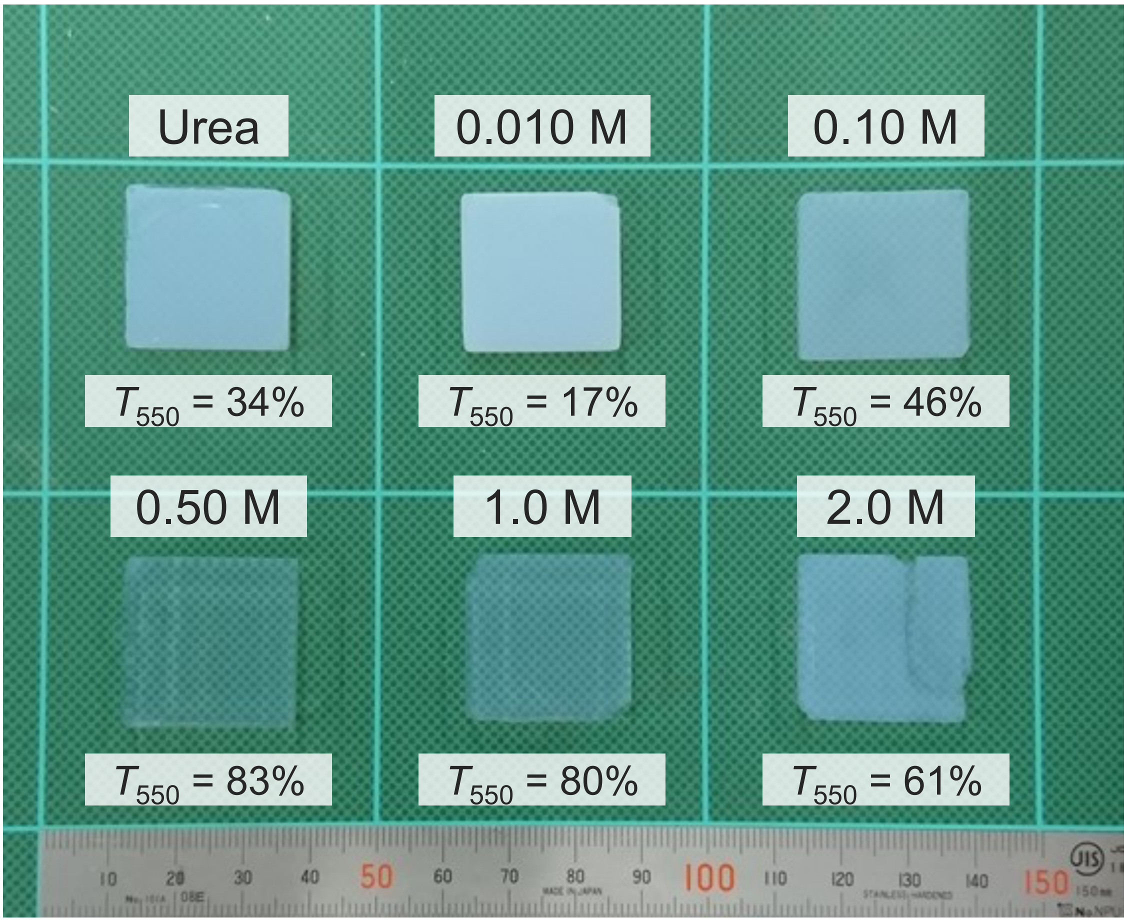

Supplementary Figure 3. Appearance of the PMSQ aerogels prepared in the presence of F127.

The base catalyst (source) for polycondensation is urea or varied concentration of TMAOH.

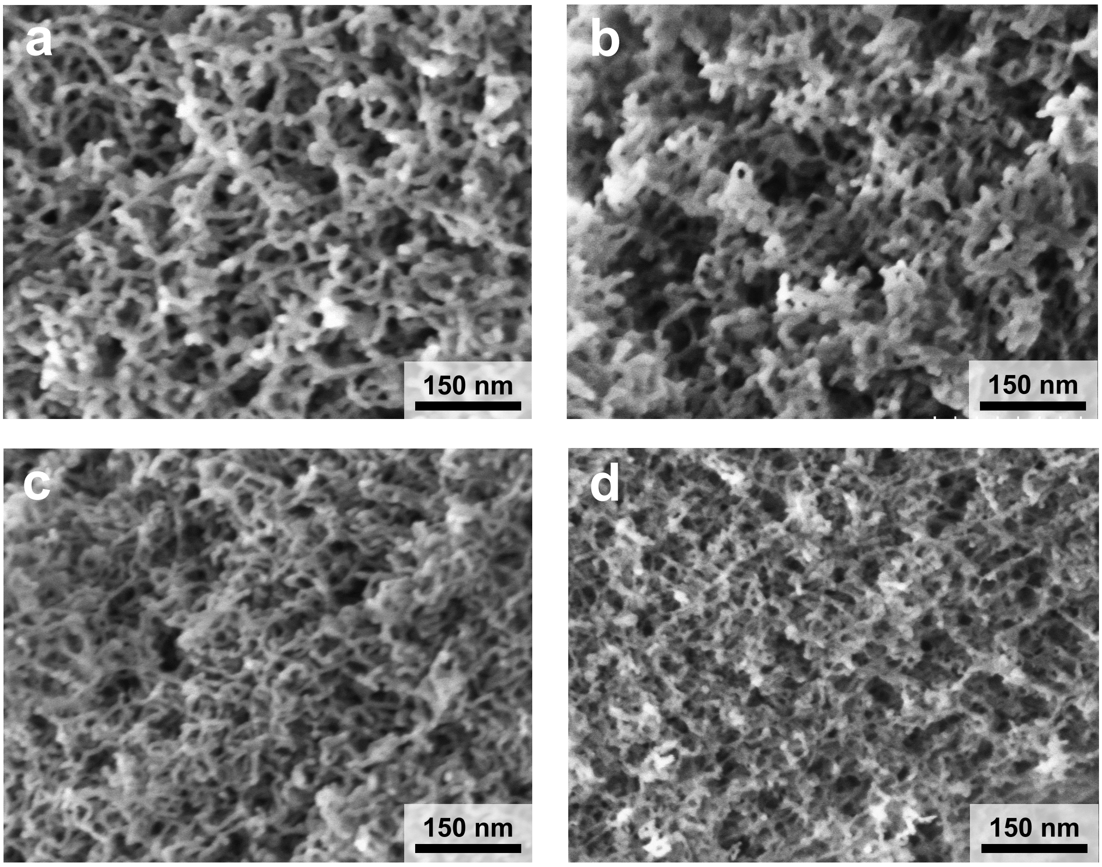

Supplementary Figure 4. FE-SEM images of the PMSQ aerogels in Supplementary Figure 3; the base (source) is (a) urea and (b) 0.010 M, (c) 0.50 M and (d) 2.0 M TMAOH.

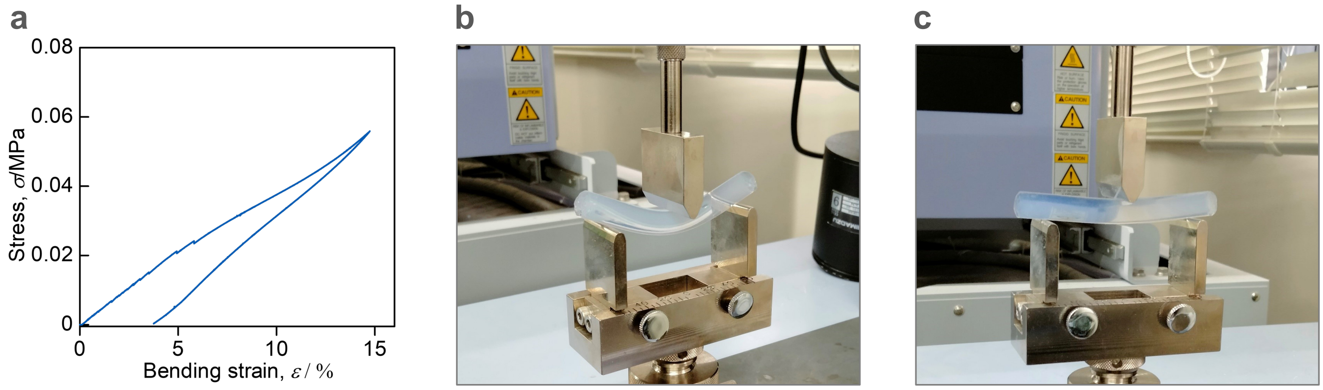

Supplementary Figure 5. Deformation and recovery of PMSQ-F127 in a single three-point bending cycle test with 60 mm span. (a) A stress–strain curve obtained by bending to 15% strain. (b, c) Appearance of PMSQ-F127 at (b) 15% bending strain and (c) the end of the cycle test.

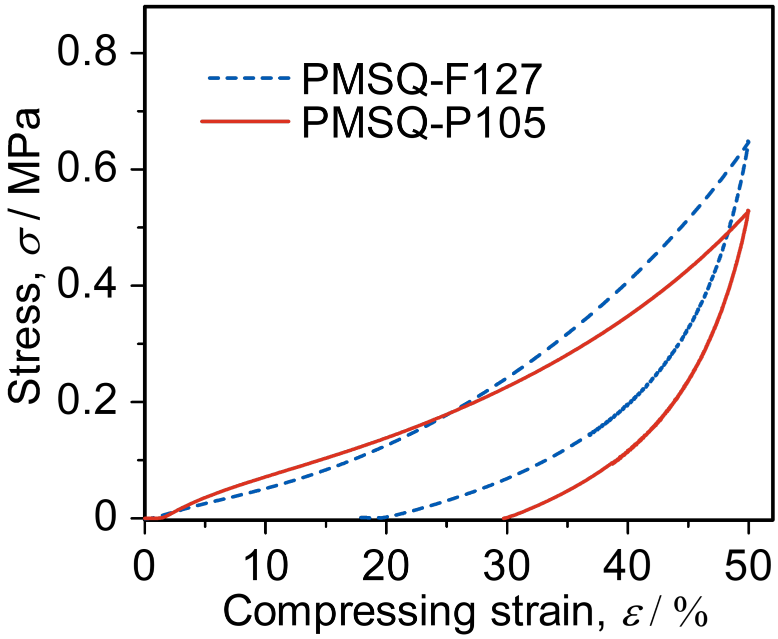

Supplementary Figure 6. Stress–strain curves obtained by a uniaxial compression–decompression test with 50% strain on PMSQ-F127 and -P105. There are some residual strains on both PMSQ-F127 and -P105 after the test.

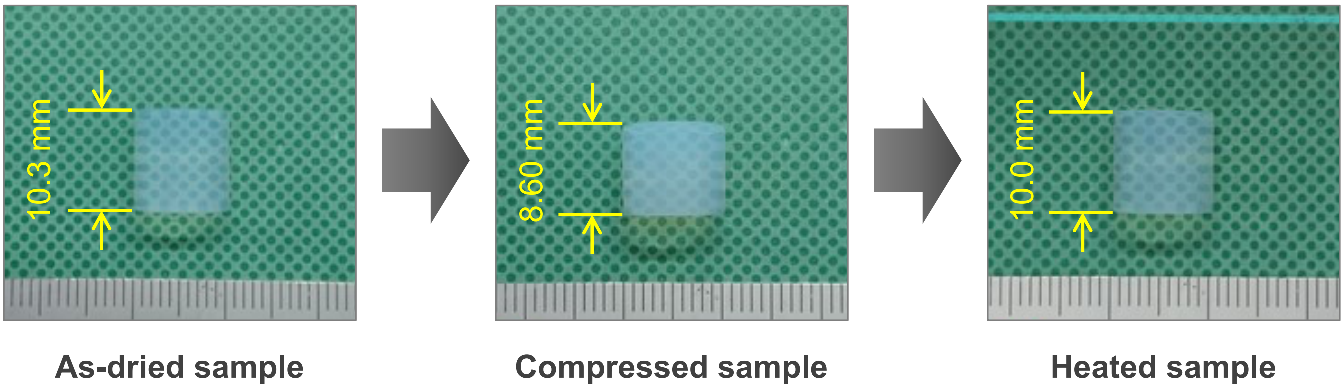

Supplementary Figure 7. Photographs of viscoelastic behavior of PMSQ-F127 during a uniaxial compression–decompression test with 50% strain. The specimen is cylindrical, ca. 10 mm diameter and height. The height of the compressed specimen was 80% of the one before the test (from 10.3 to 8.60 mm). However, the compressed specimen almost recovered its original size (10.0 mm height) by heating at 110 °C for 2 h.

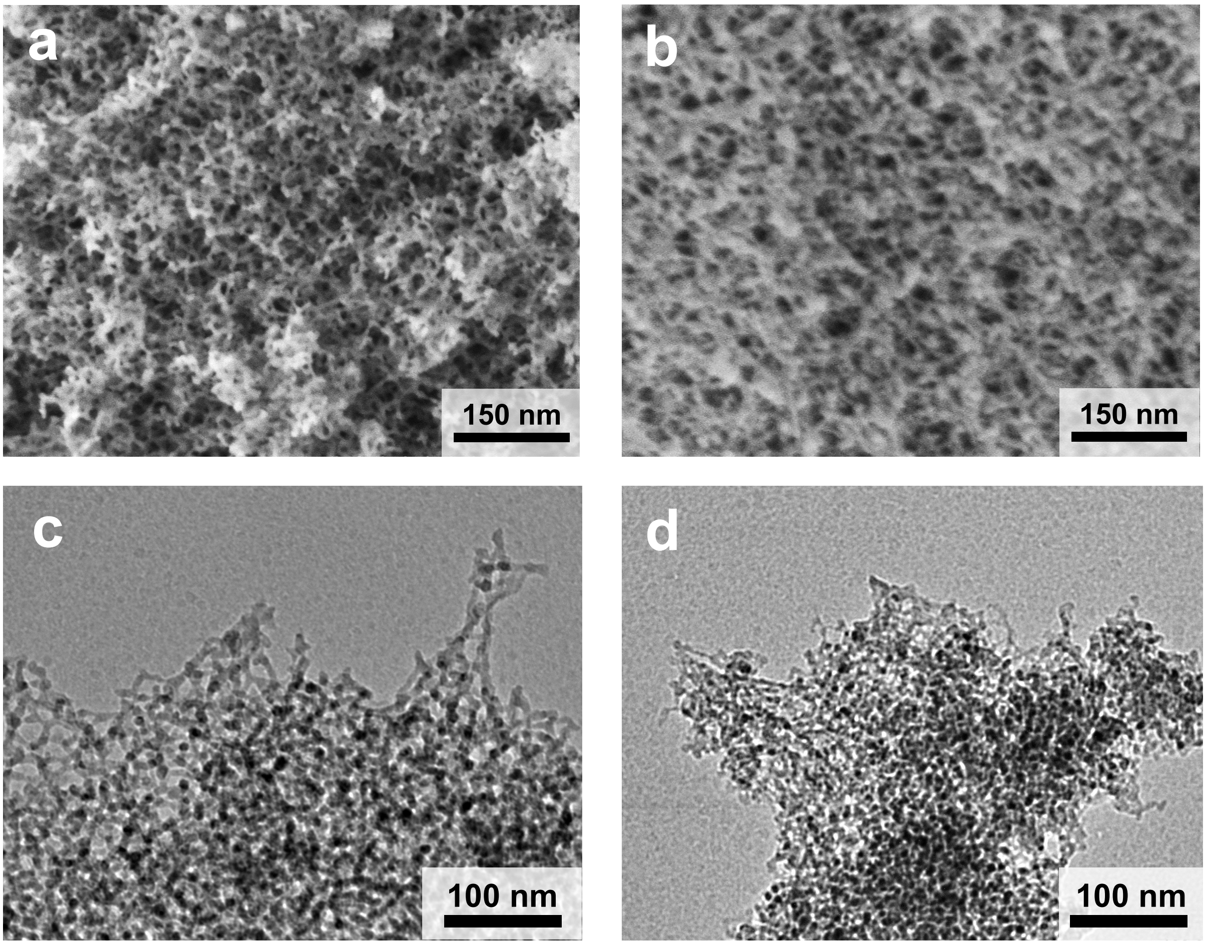

Supplementary Figure 8. (a, b) FE-SEM and (c, d) TEM images of the pore structures of PMSQ-prev and -P105DMF. (a, c) PMSQ-prev; (b, d) PMSQ-P105DMF.

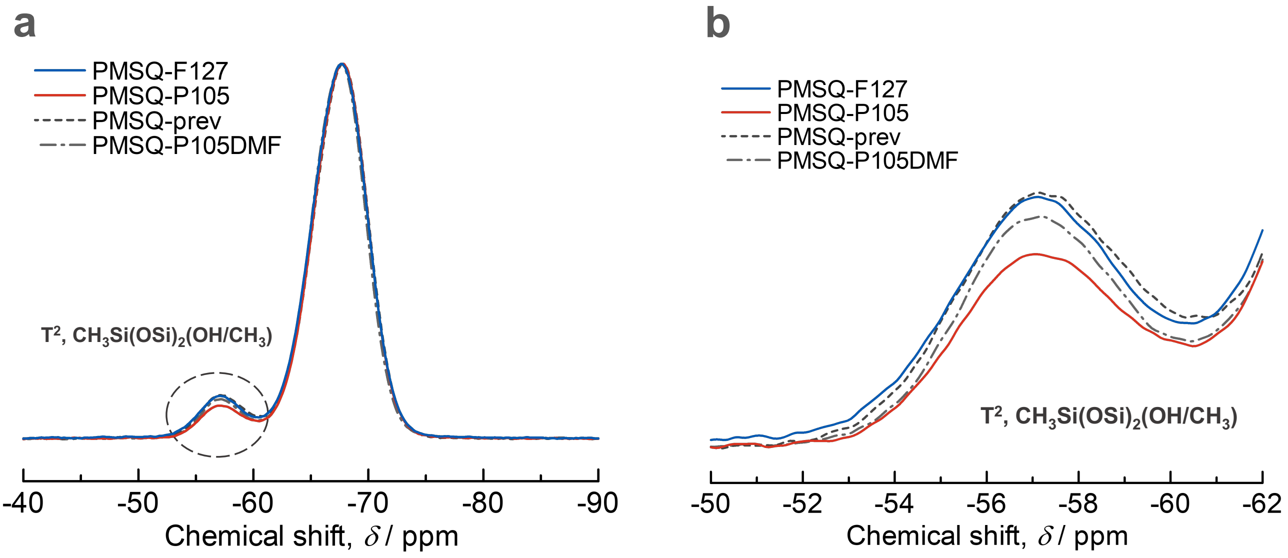

Supplementary Figure 9. ^{29}Si CP/MAS NMR spectra of the PMSQ aerogel samples. Note that the intensity of the spectra is normalized by the T^3 peak intensities. Spectra of (a) all measured chemical shift range and (b) magnified T^2 region.

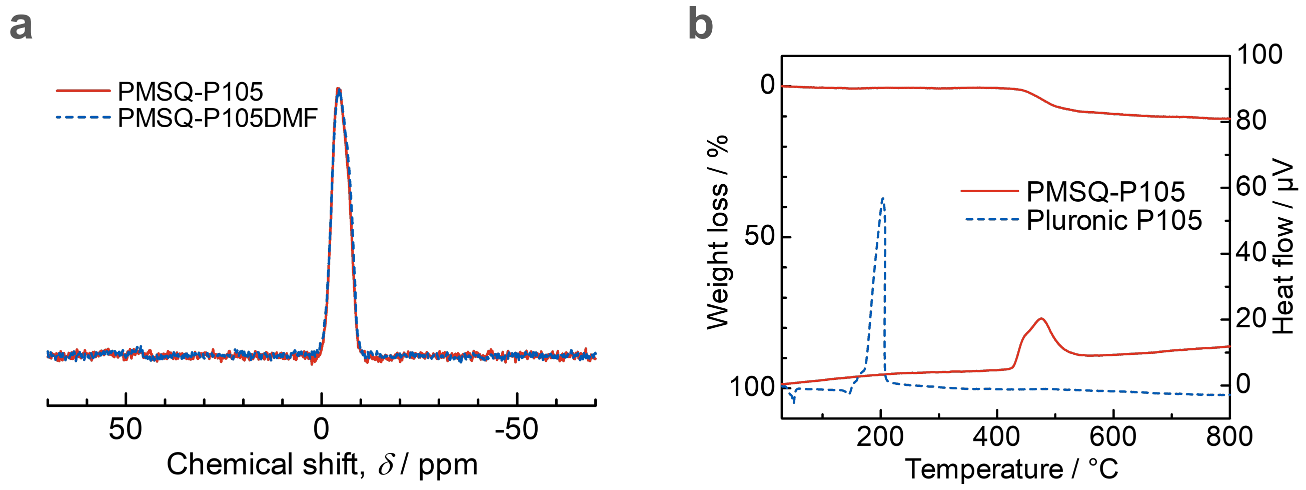

Supplementary Figure 10. Estimation of residual surfactants in the PMSQ aerogels. (a) ^{13}C CP/MAS NMR spectra of PMSQ-P105 and -P105DMF. (b) TG and DTA curves of the aerogel PMSQ-P105 and the surfactant P105. No residual surfactant was detected in either measurement.

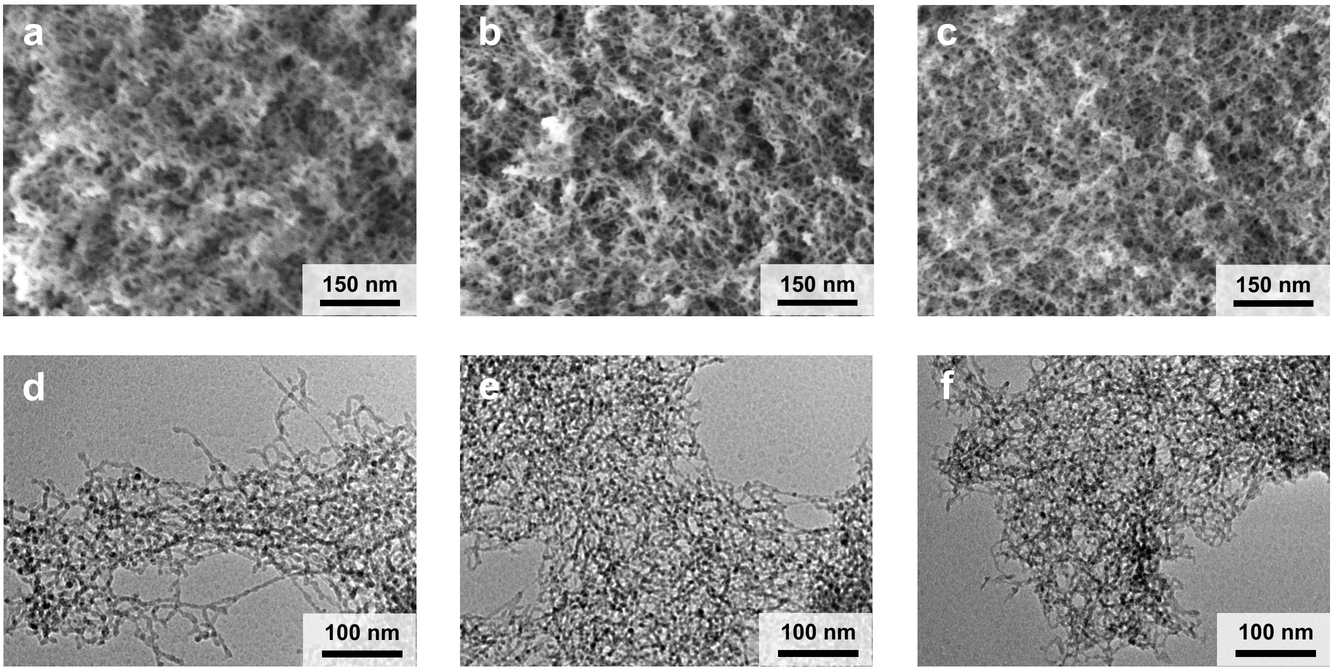

Supplementary Figure 11. (a–c) FE-SEM and (d–f) TEM images of the pore structures of PMSQ-F68, -P94, and -L64. (a, d) PMSQ-F68; (b, e) PMSQ-P94; (c, f) PMSQ-L64.

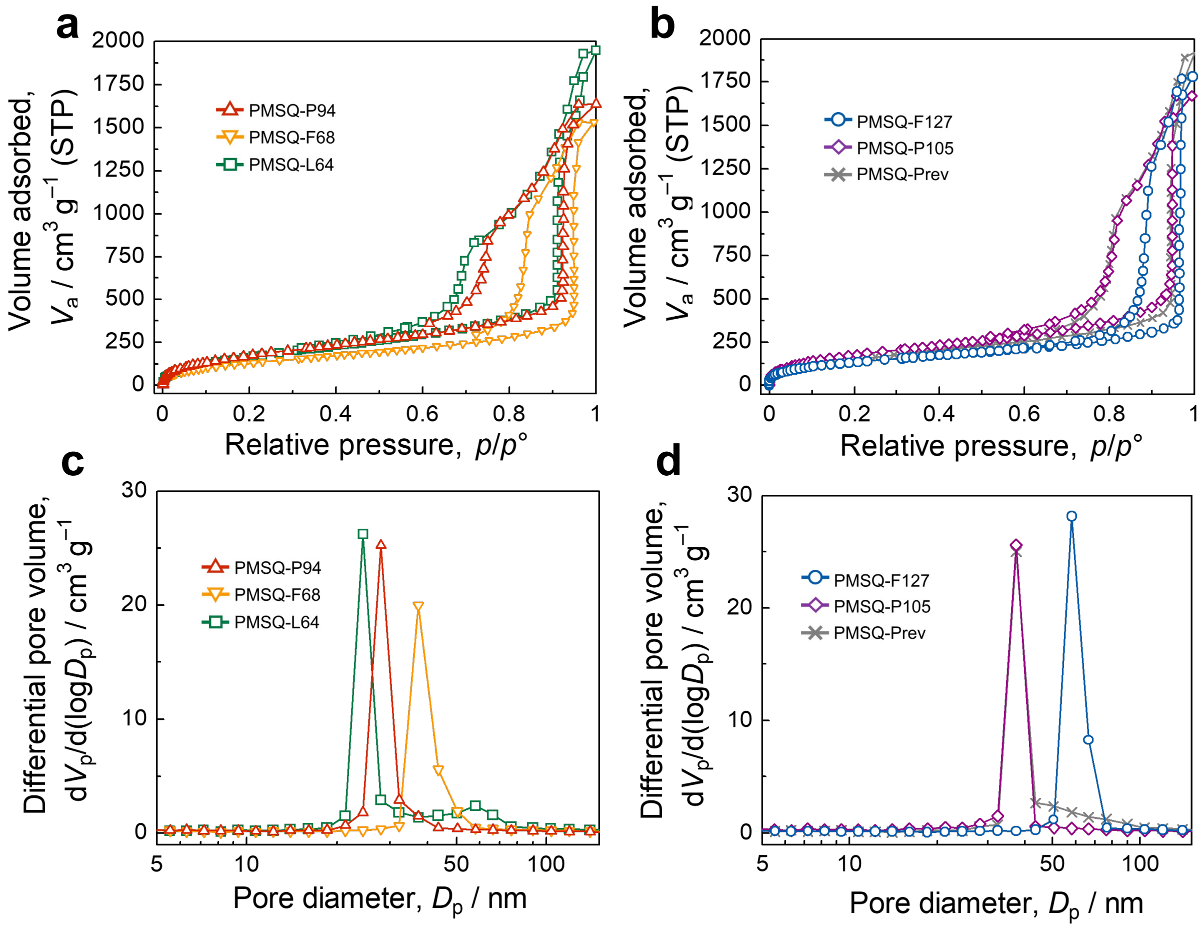

sample	BET surface area /m^2 g^{-1}
PMSQ-F127	479
PMSQ-P105	634
PMSQ-F68	504
PMSQ-P94	680
PMSQ-L64	674
PMSQ-prev	591

Supplementary Figure 12. (a, b) Nitrogen adsorption–desorption isotherms and (c, d) BJH pore size distributions of the samples. Inserted table is BET surface area of the samples derived from the nitrogen sorption isotherms at 77 K.

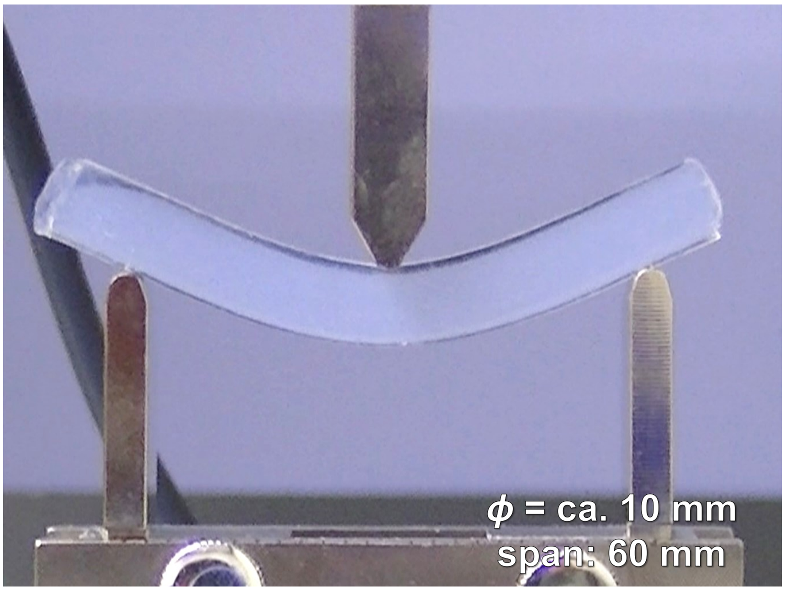

Supplementary Figure 13. A photograph of the three-point bending test on PMSQ-F68. The bent specimen in the photograph is just before the failure.

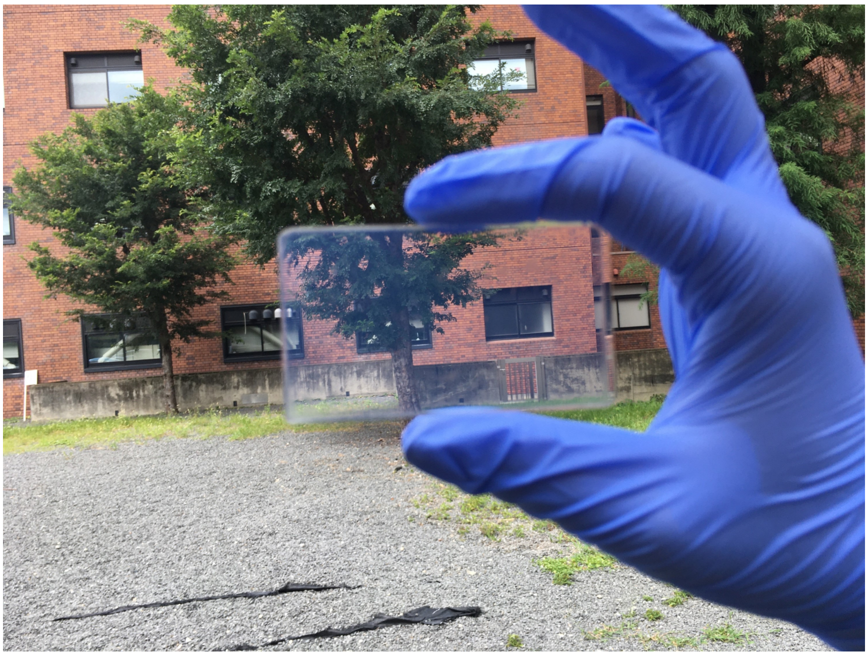

Supplementary Figure 14. A photograph of PMSQ-P94 taken outdoor to show the potential as a window insulating material. The scenery behind the aerogel can be seen clearly because of the low haze of the sample.

Supplementary Table 1. Structure and properties of PEO-*b*-PPO-*b*-PEO-type triblock copolymers employed in this study.

Surfactant	Molecular structure	Molecular weight	HLB value
F127	$EO_{106}PO_{70}EO_{106}$	12 600	18–23
P105	$EO_{37}PO_{56}EO_{37}$	6 500	12–18
F68	$EO_{76}PO_{29}EO_{76}$	8 400	> 24
P94	$EO_{26}PO_{48}EO_{26}$	5 000	13.5
L64	$EO_{13}PO_{30}EO_{13}$	2 900	12–18

Supplementary Table 2. Starting compositions of PMSQ aerogels prepared with different surfactants.

sample	MTMS/mL	5 mM HOAc/mL	Surfactant/g	Water/mL	0.50 M TMAOH/mL
PMSQ-F127	5.0	5.0	1.1	4.0	3.0
PMSQ-P105	5.0	5.0	2.7	4.0	3.0
PMSQ-F68	5.0	5.0	1.5	4.0	3.0
PMSQ-P94	5.0	5.0	3.25	4.0	3.0
PMSQ-L64	5.0	5.0	3.15	0.0	7.0

Supplementary Table 3. Size parameters of the pore structures of the PMSQ aerogels.*

Sample	Skeleton length /nm	Node size /nm	Skeleton thickness /nm	Pore size /nm	Skeletal ratio
PMSQ-F127	28.1±3.1	11.2±0.5	8.0±0.4	16.4±1.4	3.5±0.6
PMSQ-P105	25.3±2.0	9.2±0.5	6.6±0.3	16.4±1.4	3.8±0.5
PMSQ-F68	16.4±1.3	10.0±0.4	5.6±0.4	11.4±0.8	2.9±0.4
PMSQ-P94	15.8±1.3	8.2±0.4	4.2±0.4	9.0±0.6	3.8±0.7
PMSQ-L64	9.3±0.8	7.5±0.3	9.8±1.8	5.3±0.4	0.95±0.3
PMSQ-prev	11.6±0.8	9.6±0.5	6.2±0.4	10.8±0.8	1.9±0.2
PMSQ-P105DMF	16.5±1.3	13.4±0.7	9.6±1.8	14.4±1.3	1.7±0.5

*All these data are average values with a 95% confidence interval, which were obtained by digitally measuring on TEM (30 points) and FE-SEM (50 points) images of these samples using a software "Click Measure". Skeleton thickness was calculated from TEM images, and the others were from FE-SEM images. The error of skeletal ratio is the maximum error estimated from the errors of skeleton length and skeleton thickness. Definitions of each parameter are given in Supplementary Figure 15.

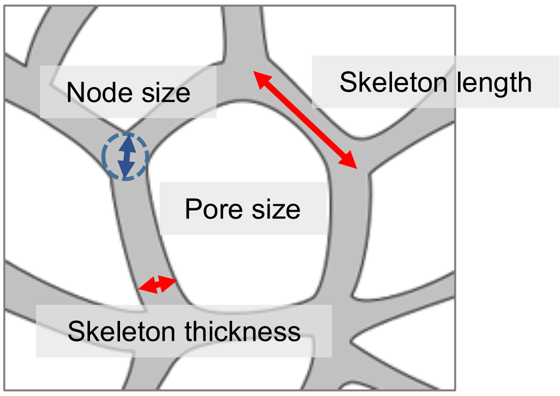

Supplementary Figure 15. Definitions of the size parameters. Node size is the diameter of an inscribed circle at a node. Skeleton length is the distance between the centers of two neighbor nodes.

Pore size is the maximum length of a pore. Skeletal ratio is defined as (skeleton length)/(skeleton thickness).
