# Supplementary material for: Unusual flexibility of transparent poly(methylsilsesquioxane) aerogels by surfactant-induced mesoscopic fiber-like assembly
Source: Nat Commun. 2024 Jan 11;15:461. doi: 10.1038/s41467-024-44713-5 (PMC10784555; doi:10.1038/s41467-024-44713-5)
Supplement: Supplementary file 3 — Description of additional supplementary files [file 41467_2024_44713_MOESM3_ESM.pdf]

Description of additional supplementary files

Supplementary Movie 1: Bendability of PMSQP105 in the three-point bending test.

Supplementary Movie 2: Bendability of PMSQF127 in the three-point bending test.
